# Rational Design of 3D Honeycomb-Like SnS_2_ Quantum Dots/rGO Composites as High-Performance Anode Materials for Lithium/Sodium-Ion Batteries

**DOI:** 10.1186/s11671-018-2805-x

**Published:** 2018-12-03

**Authors:** Yingge Zhang, Yan Guo, Yange Wang, Tao Peng, Yang Lu, Rongjie Luo, Yangbo Wang, Xianming Liu, Jang-Kyo Kim, Yongsong Luo

**Affiliations:** 1 School of Physics and Electronic Engineering, Xinyang Normal University, Xinyang, 464000 People’s Republic of China; 2 Key Laboratory of Microelectronics and Energy of Henan Province, Xinyang Normal University, Xinyang, 464000 People’s Republic of China; 3 College of Chemistry and Chemical Engineering, Luoyang Normal University, Luoyang, 471934 People’s Republic of China; 4 Department of Mechanical and Aerospace Engineering, Hong Kong University of Science and Technology, Clear Water Bay, Kowloon, Hong Kong, People’s Republic of China

**Keywords:** SnS_2_ quantum dots, Spray drying, rGO, Lithium-ion batteries, Sodium-ion batteries

## Abstract

Structure pulverization and poor electrical conductivity of metal dichalcogenides result in serious capacity decay both in lithium-ion batteries (LIBs) and sodium-ion batteries (SIBs). To resolve the above problems, a combination of metal dichalcogenides with conductive scaffolds as high-performance electrode materials has aroused tremendous interest recently. Herein, we synthesize a 3D honeycomb-like rGO anchored with SnS_2_ quantum dots (3D SnS_2_ QDs/rGO) composite via spray-drying and sulfidation. The unique 3D-ordered honeycomb-like structure can confine the volume change of SnS_2_ QDs in the lithiation/delithiation and sodiation/desodiation processes, provide enough space for electrolyte reservoirs, promote the conductivity of the SnS_2_ QDs, and improve the electron transfer. As a result, the 3D SnS_2_ QDs/rGO composite electrode delivers a high capacity and long cycling stability (862 mAh/g for LIB at 0.1 A/g after 200 cycles, 233 mAh/g for SIB at 0.5 A/g after 200 cycles). This study provides a feasible synthesis route for preparing 3D-ordered porous networks in varied materials for the development of high-performance LIBs and SIBs in future.

**Electronic supplementary material:**

The online version of this article (10.1186/s11671-018-2805-x) contains supplementary material, which is available to authorized users.

## Background

Energy storage plays a remarkable role in modern life. Li-ion batteries (LIBs) have been widely applied as a power source for portable electronic devices and power electric vehicles due to their long cycle life stability and high energy density. Meanwhile, Na-ion batteries (SIBs) have attracted tremendous attention in renewable energy storage because of their low cost and environmental benignity. While the commercial graphite anodes of LIBs show low theoretical capacity (372 mAh/g) and suffer from structural instability and safety problem during a high-rate charge-discharge process, also cannot be used in SIBs owing to their small inter-layer space [1–4]. Therefore, novel anode materials with high capacity need to be developed for the next-generation LIBs and SIBs.

Metal dichalcogenides, possessing high theoretical capacities, are promising candidates for replacing the commercial graphite in LIB and SIB applications. Among the metal dichalcogenides, layered SnS_2_ exhibits a higher theoretical capacity than graphite, and has been regarded as an attractive anode material. SnS_2_ is a typical CdI_2_-type, where the each layer connects with each other mainly by weak Van der Waals force. Such a feature makes it a desired intercalation/deintercalation candidate for Li^+^ and Na^+^ in the first step of the conversion reaction. However, SnS_2_ suffers from large volume change and poor electrical conductivity in the charge-discharge process, thus results in severe capacity decay.

Integrating SnS_2_ with other conductive scaffolds to design a rational structure especially a three-dimensional (3D)-ordered porous network has been considered as feasible strategies to improve the electrical conductivity and cycling stability of LIBs and SIBs [5, 6]. Graphene is considered a promising candidate for scaffolds owing to its excellent mechanical characteristics and electronic conductivity. Firstly, compared to 1D and 2D structures, a 3D-ordered porous network is more conducive to the complete contact between the electrode and electrolyte. Thus, it can act as a channel for fast electron transport along the 3D direction and restrain the aggregation effectively [7]. Secondly, the rich pores in 3D-ordered porous network can relieve the volume expansion in three dimensions space and thus makes it exhibit long cycle life stability [8–14]. Zhu et al. designed Co_3_O_4_ with a 3D mesoporous network and showed excellent performance in LIBs [15]. Deng et al. demonstrated a new 3D-ordered macroporous MoS_2_/carbon nanostructure is beneficial for obtaining high-performance of LIBs [16]. Choi et al. synthesized layered WS_2_ nanosheet-decorated 3D-RGO microspheres as an anode material for SIBs [17]. Based on the above discussion, we have designed a unique 3D honeycomb-like structure to buffer the large volume change and enhance the electrical conductivity of SnS_2_ by spray drying and sulfidation. The composite achieves excellent electrochemical performance in both LIBs and SIBs (862 mAh/g for LIB at 0.1 A/g after 200 cycles, 233 mAh/g for SIB at 0.5 A/g after 200 cycles).

The 3D-structured honeycomb-like rGO anchored with SnS_2_ quantum dots composite (3D SnS_2_ QDs/rGO) via a two-step method. Firstly, the 3D honeycomb-like rGO anchored with SnO_2_ composite (3D SnO_2_/rGO) is synthesized through spray drying and post-calcination. Then, it is annealed with thiourea to obtain the 3D SnS_2_ QDs/rGO composite under the argon atmosphere. The 3D honeycomb-like structure can reduce the inter-sheet junction contact resistance effectively, provide a large accessible active surface area for the adsorption/desorption of ions, restrain the aggregation of SnS_2_ QDs, and buffer the volume expansion of SnS_2_ QDs [18–20]. As a result, the SnS_2_ QDs with a diameter of ~ 6 nm are uniformly distributed within the rGO layer after 200 charge/discharge cycles in the LIB test. Moreover, the 3D SnS_2_ QDs/rGO composite electrode possesses a high capacity and long cycling stability (862 mAh/g for LIB at 0.1 A/g after 200 cycles, 233 mAh/g for SIB at 0.5 A/g after 200 cycles). The unique metal sulfide-based 3D porous graphene materials presented in this study provide a way to the development of high-performance LIBs and SIBs.

## Methods

### Synthesis of Polystyrene Nanospheres

All reagents used were of analytical grade and were used directly without any purification. Styrene was alternatively washed with DI water and 1 M NaOH to remove the polymer inhibitors. Then, 8 ml styrene, 92 ml DI water, and 0.2 g K_2_S_2_O_8_ were mixed and then stirred at 80 °C for 10 h in argon atmosphere. Finally, a white product was obtained by centrifugation. After washed by DI water and ethanol for at least five times, the product was freeze-dried at − 50 °C for 24 h [16].

### Fabrication of 3D SnS_2_ QDs/rGO Composite

In a typical synthesis, 24 g graphene oxide (GO) colloidal (2.5 wt%) that was obtained by the modified Hummer approach was added into 500 ml DI water. Then, 3 g polystyrene (PS) nanospheres were dispersed in the prior solution [21, 22]. Further, 1.5 g tin (IV) chloride pentahydrate (SnCl_4_^.^5H_2_O) was placed into the mixture and ultra-sonication for 1 h. The mixture solution was spray-dried with exit temperature of 140 °C and a flow rate of 800 ml/h. Subsequently, the collected product was annealed at 450 °C for 2 h at a ramping rate of 3 °C min^−1^ in Ar atmosphere to remove PS nanospheres, and then the 3D SnO_2_/rGO was obtained. Finally, thiourea, acting as the sulfur source, was mixed with the SnO_2_/rGO. And then it was annealed at 350 °C for 12 h at a heating rate of 2 °C min^−1^ in Ar atmosphere to ensure the 3D SnO_2_/rGO composite completely transformed into 3D SnS_2_ QDs/rGO composite [23]. The pure SnS_2_ composite was synthesized in the absence of GO and PS nanospheres.

### Characterization

The crystalline structure and phase of the composites were tested by X-ray diffraction (XRD, D8-Advance Bruker) with Cu-Kα (λ = 1.5418 Å) radiation at 40 kV and 40 mA, ranging from 10 to 80 °C at room temperature. The surface chemical composition of the composites was analyzed by a modified X-ray photoelectron spectroscopy (XPS, PHI 5600). The morphologies and structures were examined by field emission scanning electron microscope (FESEM, JEOL S-4800) and transmission electron microscope (TEM, JEOL JEM-2010). The Brunauer–Emmett–Teller (BET) surface area and pore size were identified by using the nitrogen adsorption/desorption isotherms obtained at 77 K on a surface area and porosity analyzer (Quadrasorb SI-MP, Quantachrome). The Raman spectrum was obtained by an INVIA Raman microprobe (Renishaw Instruments) with a 532 nm laser source and a × 50 objective lens. The thermogravimetric analyzer (TGA) curve was performed using an STD Q600 TA with 100 ml min^−1^ of air flow from 30 to 800 °C at a heating rate of 10 °C min^−1^.

### Electrochemical Test

To prepared working electrodes, 70 wt% 3D SnS_2_ QDs/rGO composite, 20 wt% acetylene black, and 10 wt% polyvinylidene fluoride were mixed and dissolved in *N*-methyl-2-pyrrolidinone. After stirring for 5 h, the obtained slurry was coated onto the copper foil (acted as a current collector) and dried at 80 °C in vacuum overnight. The electrochemical tests were carried out using two-electrode cells assembled in an argon-filled glove box. Li and Na metals acted as the counter electrode. The organic electrolyte in LIBs was constituted of 1.0 M LiPF_6_ in ethylene carbonate (EC) and diethyl carbonate (DEC) (1:1, *v*/*v*). For SIBs, the electrolyte was 1 M NaClO_4_ in a mixture of EC/DEC (1:1, *v*/*v*). Galvanostatic charge/discharge measurements were performed by a battery test system (NEWARE, Shenzhen Xinwei Electronics, Ltd) at different current densities with a voltage range of 0.01–3.00 V. Cyclic voltammetry (CV) and the cyclic voltammograms were recorded over the potential range of 0.01–3.00 V with a scan rate of 0.1 mV/s.

## Results and Discussion

**Scheme 1:**
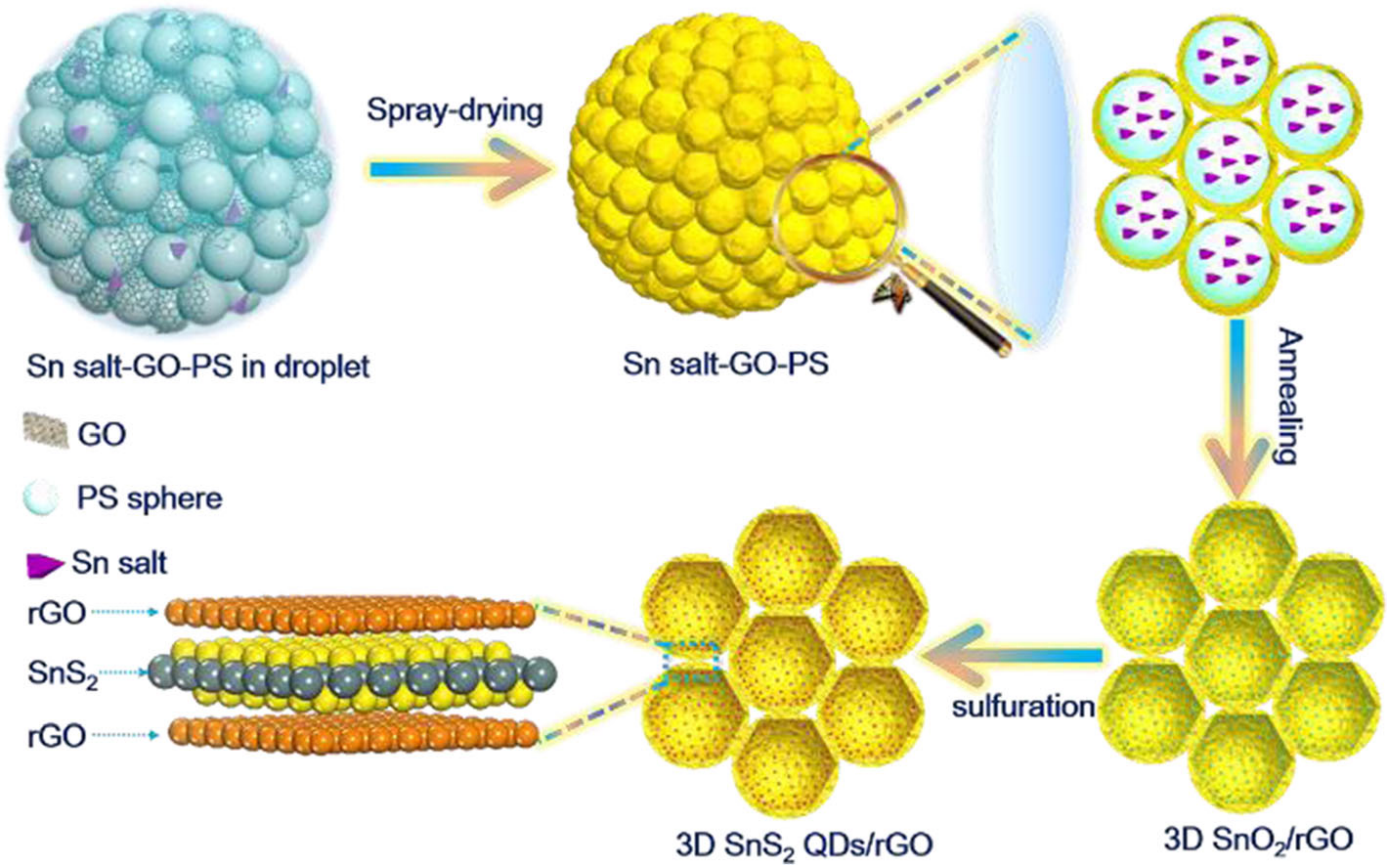
Schematic of the fabrication of the 3D SnS_2_ QDs/rGO composite by spray drying and sulfuration and the interface microstructure model of the SnS_2_/rGO composite

**Fig. 1:**
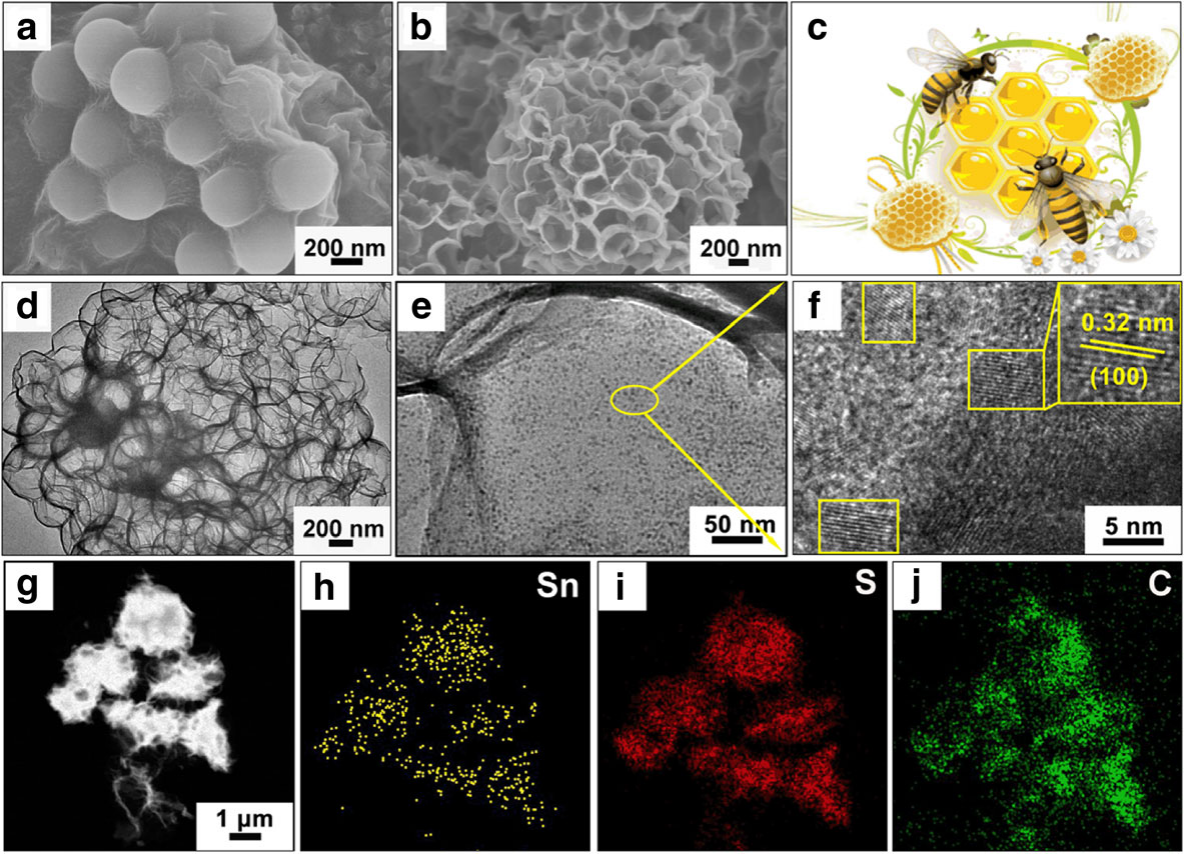
**a** SEM image of the Sn salt-GO-PS composite. **b** SEM image of the 3D SnS_2_ QDs/rGO composite. **c** Photograph of a honeycomb-like structure. **d**, **e** TEM images of the 3D SnS_2_ QDs/rGO composite. **f** HRTEM image of the 3D SnS_2_ QDs/rGO composite. **g–j** Elemental mapping images of Sn, S, and C elements

Scheme 1 illustrates the synthesis process of the 3D SnS_2_ QDs/rGO composite. A colloidal solution, consisting of uniformly dispersed GO nanosheets, PS nanospheres, and tin (IV) chloride pentahydrate is stirred for 6 h at room temperature. To ensure that no precipitate is formed, the colloidal solution is made to stand for several hours before being nebulized. Subsequently, the Sn salt-GO-PS composite is formed inside the reactor in 10 s (Fig. 1a). Second, the 3D SnO_2_/rGO composite is synthesized through calcination in Ar atmosphere, as shown in Additional file 1: Figures S1a and S1b. During the formation of the 3D SnO_2_/rGO composite, the PS nanospheres with a mean size of 200–300 nm act as a sacrificed template is uniformly anchored on the rGO layers. After calcination, the decomposition of the PS nanospheres results in 200–300-nm-sized voids, lead to the formation of a 3D honeycomb-like structure, as shown in Additional file 1: Figure S1c. Finally, thiourea is used as the sulfur source and reductant to react with the precursor 3D SnO_2_ /rGO to obtain honeycomb-like 3D SnS_2_ QDs/rGO composite (Fig. 1b, c). The TEM image in Fig. 1d further demonstrates the 3D honeycomb-like structure, which is consistent with the morphology presented in the SEM images. Moreover, the thinned layers of rGO nanosheets of the 3D SnS_2_ QDs/rGO composite can be clearly observed in the TEM image shown in Additional file 1: Figure S1d. The ultrafine SnS_2_ QDs with several nanometers in size is distributed within the 3D rGO layers while compared Figs. 1e, f with Additional file 1: Figure S1d. The enlarged TEM image of the SnS_2_ QDs showed in Fig. 1f indicates clear lattice fringes separated by 0.32 nm, which correspond to the (100) plane of SnS_2_. The distribution of Sn, S, and C in the composite was uniform as shown in Fig. 1g–j.

**Fig. 2:**
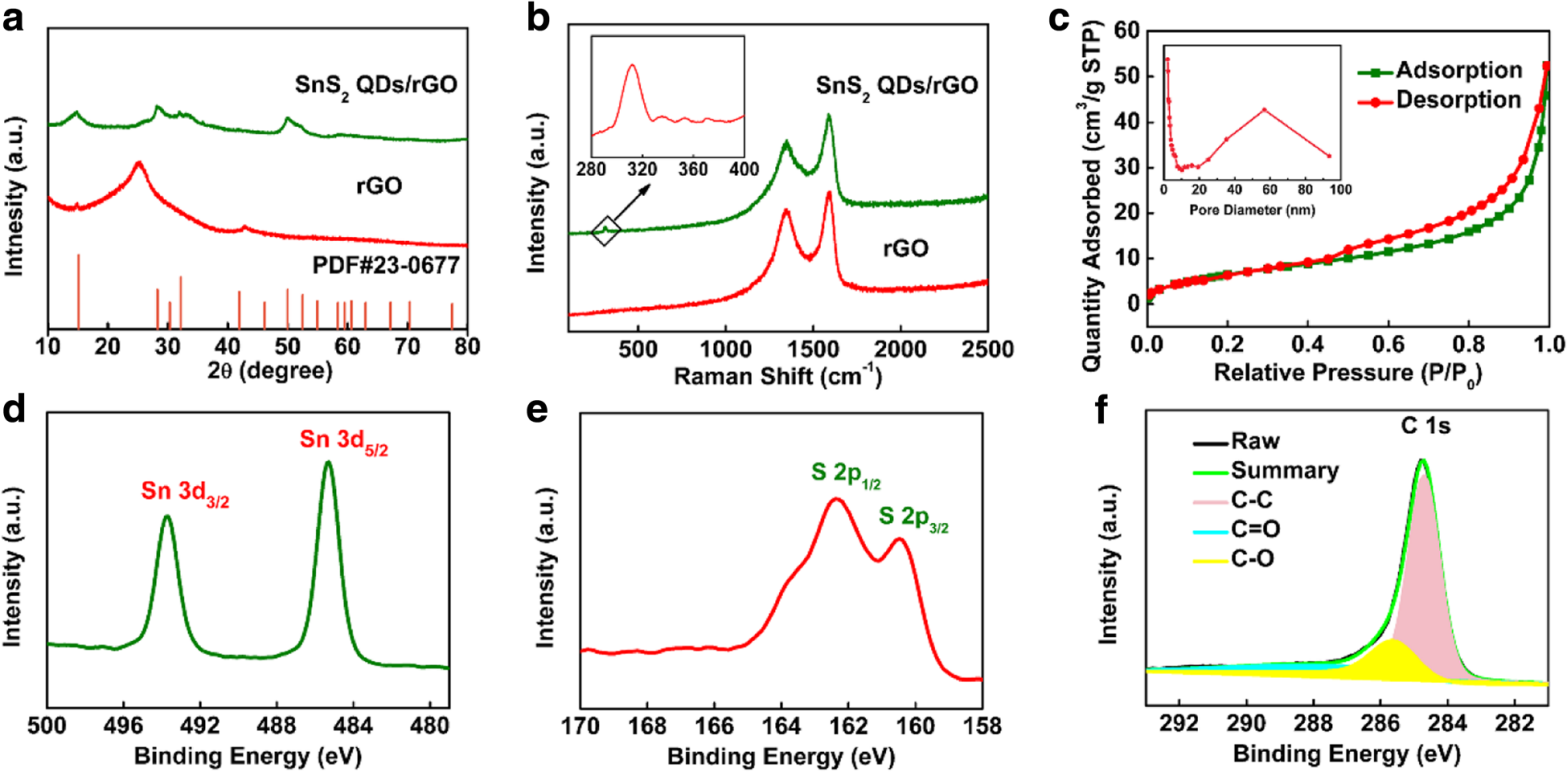
**a** XRD pattern and **b** Raman spectra of the rGO and 3D SnS_2_ QDs/rGO composite. **c** N_2_ adsorption–desorption isotherms and the corresponding pore size distributions of the 3D SnS_2_ QDs/rGO composite. High-resolution XPS spectra of **d** Sn 3d, **e** S 2p, and **f** C 1 s of the 3D SnS_2_ QDs/rGO composite

The XRD patterns of rGO and the 3D SnS_2_ QDs/rGO composite are shown in Fig. 2a. The rGO presents three diffraction peaks at 2θ = 15.04°, 26.14°, and 44.52°. The first peak belongs to the characteristic peak of GO, which is further verified by the following Raman spectrum. The next two peaks are attributed to the (002) and (100) lattice planes of the hexagonal graphene (JCPDS No. 03-065-2023). The diffraction peaks of 3D SnS_2_ QDs/rGO can be observed at 15.0°, 28.2°, 30.26°, 41.9°, 49.96°, 58.35°, and 70.33°, which correspond to the crystal planes (001), (100), (002), (102), (110), (200), and (113) (JCPDS No. 23-0677) of SnS_2_, respectively [24]. Compared to the pure SnS_2_ showed in Additional file 1: Figure S2a, the relatively broad diffraction peaks of the 3D SnS_2_ QDs/rGO composite indicate smaller particle sizes, which are in accordance with the TEM results. To further investigate the structure of the 3D SnS_2_ QDs/rGO composite, the Raman spectrums of the composite and rGO are obtained in Fig. 2b. The Raman peaks of rGO that appeared at 1596 and 1348 cm^−1^ are attributed to the G and D bands of the carbon structure, respectively. Generally, the D band is relevant to the defects of carbon atoms in graphitic layers, while the G band belongs to the stretching vibration of -C=C- in a 2D hexagonal lattice. A much weaker peak appeared at about 309 cm^−1^ in the 3D SnS_2_ QDs/rGO composite, which corresponds to the characteristic peak of the A_1g_ mode of the SnS_2_ phase [25]. Moreover, the D band observed at 1349 cm^−1^ and the G band observed at 1587 cm^−1^ belonged to 3D SnS_2_ QDs/rGO [26]. While the SnS_2_ can influence the reduction in GO and hinder its reduction, the composite exhibits a slightly higher intensity in D peak than rGO [27]. Such result can also explain the peak that appears at 2θ = 15.04° in XRD patterns. To investigate the BET surface area and pore size, the internal porosity and microstructure of the as-prepared 3D SnS_2_ QDs/rGO composite are measured by nitrogen adsorption- desorption measurements. The remarkable hysteresis loops of N_2_ adsorption-desorption isotherms shown in Fig. 2c can be assigned to the type IV loop, which demonstrates the standard nanoporous structure of the composite. The specific surface area of the composite is calculated to be 21.99 m^2^ g^−1^ by using a multi-point BET method according to the adsorption branch of the isotherm. As a consequence, the 3D SnS_2_ QDs/rGO composite with such a pore structure can provide more active sites and is conducive to the ion diffusion in the charge/discharge process [28, 29].

The surface chemical composition and oxidation state of the composite are analyzed by XPS system. In Fig. 2d, the two prominent peaks at 487.3 and 495.7 eV are ascribed to Sn 3d_3/2_ and Sn 3d_5/2_, respectively. The energy difference between Sn 3d_5/2_ and Sn 3d_3/2_ is 8.4 eV, which indicates the Sn^4+^ oxidation state [30]. The survey XPS S 2p spectrum is presented in Fig. 2e. The characteristic peaks appearing at 161.3 and 163.4 eV are attributed to S 2p_3/2_ and S 2p_1/2_ for S^2−^ in SnS_2_ [27, 31]. The XPS spectrum of C 1s showed in Fig. 2f can be fitted and split into three different peaks of 284.7, 285.7, and 288.1 eV, respectively. The three peaks belonged to the C-C, C-O, and C=O bonds, respectively [25, 32].

The mass percentage of SnS_2_ in 3D SnS_2_ QDs/rGO composite was conducted by TGA from 30 to 800 °C at a heating rate of 10 °C/min in air. In Additional file 1: Figure S4a, the 3D SnS_2_ QDs/rGO composite was completely oxidized to SnO_2_ over 800 °C, producing a total weight loss of about 29.5%. The process of weight loss contained three processes, namely the desorption of water molecules (1.4%) adsorbed on the 3D SnS_2_ QDs/rGO composite, oxidization of SnS_2_, and the successively burning of rGO. The weight percentages of SnS_2_ in the 3D SnS_2_ QDs/rGO composite can be calculated to be 83.7%, based on the complete weight loss of rGO combustion and the partial weight loss from the transformation of SnS_2_ into SnO_2_ [28].

**Fig. 3:**
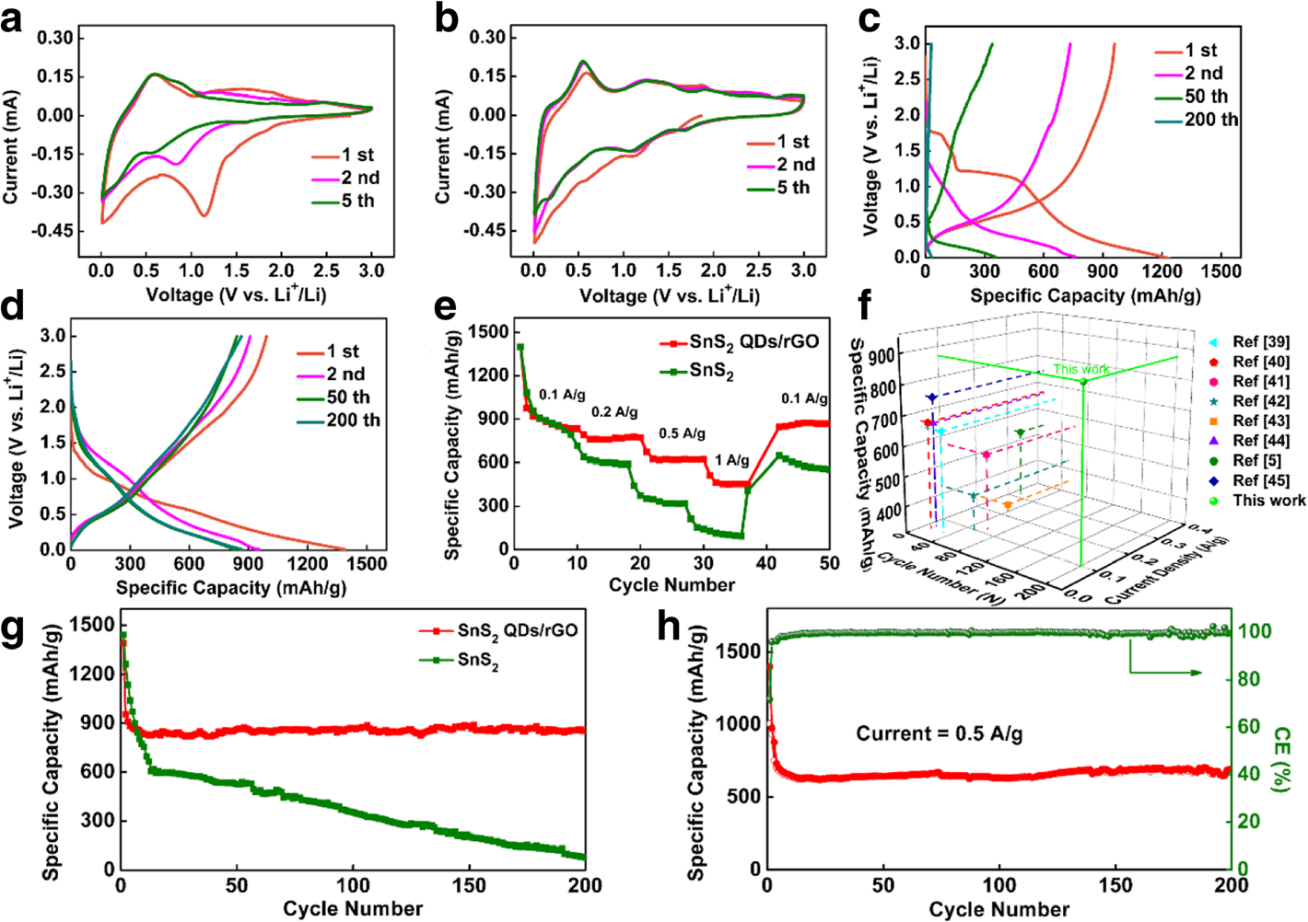
Electrochemical performance of the pure SnS_2_ and 3D SnS_2_ QDs/rGO composite electrodes for LIBs: **a**, **b** CV curves of the pure SnS_2_ and 3D SnS_2_ QDs/rGO composite electrodes at a scan rate of 0.1 mV/s for the first five cycles. **c** Charge/discharge curves of the pure SnS_2_ composite electrode and **d** the 3D SnS_2_ QDs/rGO composite electrode at a current density of 0.1 A/g in the voltage range of 0.01–3.0 V vs. Li^+^/Li. **e** Rate performance of the 3D SnS_2_ QDs/rGO and pure SnS_2_ composite electrodes at rates ranging from 0.1 to 1 A/g. **f** Comparison of electrochemical performance between the 3D SnS_2_ QDs/rGO composite (the current study) and previously reported SnS_2_-based material composite. **g** Cycling performances of 3D SnS_2_ QDs/rGO and pure SnS_2_ composite electrodes at a current density of 0.1 A/g. **h** Cycling performance and Coulombic efficiency of 3D SnS_2_ QDs/rGO composite electrode at a current density of 0.5 A/g

To investigate the lithium storage processes of the 3D SnS_2_ QDs/rGO and the pure SnS_2_ as anode materials for LIBs, their CV curves are tested at a scan rate of 0.1 mV/s, as shown in Fig. 3a, b. In Fig. 3a, the reduction peaks at 1.0–1.5 V are attributed to phase decomposition, structure collapse, and formation of a solid electrolyte interface (SEI) layer. In Fig. 3b, the first reduction peak at 1.7 V is assigned to the intercalation of Li^+^ into the SnS_2_ nanostructure during the first cycle [33]. The second reduction peak at 1.1 V is attributed to the decomposition of SnS_2_ QD to metallic Sn and Li_2_S (as shown in reaction (1)) [34]. The third reduction peak below 0.5 V indicates the appearance of Li_x_Sn alloys according to reaction (2) and the Li^+^ inserted into the rGO layered nanostructure [35, 36]. During reverse scanning, the first oxidation peak at 0.52 V indicates the de-alloying of Li_x_Sn according to reaction (2). The second oxidation peak at 1.8 V can be attributed to the fact that the Li_2_S can decompose partly and the Sn can be oxidated to Sn^4+^ (see the reverse reaction (1)) [34, 37, 38]. The reactions of the above mentioned are as follows:

1 $$ \mathrm{Sn}{\mathrm{S}}_2+4{\mathrm{Li}}^{+}+4\ {\mathrm{e}}^{-}\to 2{\mathrm{Li}}_2\mathrm{S}+\mathrm{Sn} $$

2 $$ \mathrm{Sn}+\mathrm{x}\ {\mathrm{Li}}^{+}+\mathrm{x}{\mathrm{e}}^{-}\leftrightharpoons {\mathrm{Li}}_x\mathrm{Sn}\ \left(0\le \mathrm{x}<4.4\right) $$

Note that the intensities of the reduction peaks of SnS_2_ decrease drastically in the second and fifth scans. In contrast, the reduction peaks of the 3D SnS_2_ QDs/rGO electrode perfectly overlap in the second and fifth scans, suggesting its excellent electrochemical reversibility and stability.

Galvanostatic charge/discharge measurements of the pure SnS_2_ and 3D SnS_2_ QDs/rGO electrodes are also performed at a current density of 0.1 A/g between 0.01 and 3.00 V vs Li^+^/Li. The charge/discharge curves (1st, 2nd, 50th, and 200th cycles) are shown in Fig. 3c, d, respectively. In Fig. 3c, the charge/discharge curve of the pure SnS_2_ electrode shows a drastic decrease to 16 mAh/g after the 200th cycle. In Fig. 3d, the initial discharge capacity for the 3D SnS_2_ QDs/rGO electrode is 1400 mAh/g. It is higher than the theoretical storage capacity of Li^+^ (1231 mAh/g) of SnS_2_ calculated from both reactions (1) and (2) according to the Faraday equation. This is ascribed to the formation of a SEI layer on the surface of the 3D SnS_2_ QDs/rGO electrode caused by the irreversible insertion of Li^+^ and the decomposition of the electrolyte [3]. Upon increasing the cycles to 2, 50, and 200, the capacities of the 3D SnS_2_ QDs/rGO electrode are maintained at 975, 867, and 870 mAh/g, respectively. Obviously, the 3D SnS_2_ QDs/rGO electrode possesses an excellent charge/discharge stability and long cycle life than the pure SnS_2_ electrode.

The rate performances of the electrodes are presented in Fig. 3e. It can be seen that the discharge capacity at a rate of 0.1, 0.2, 0.5, and 1 A/g is 870, 770, 622, and 452 mAh/g, respectively. Then it easily return to 867 mAh/g at 0.1 A/g, indicating that the 3D SnS_2_ QDs/rGO composite can bear gradual rate variations and possesses remarkable electrochemical stability and reversibility. While the pure SnS_2_ electrode’ capacity decays to 792, 587, 319, and 106 mAh/g with the discharge/charge rates increased to 0.1, 0.2, 0.5, and 1 A/g, respectively. And it only restores to 662 mAh/g when the discharge/charge rate is recovered to 0.1 A/g. The outstanding electrochemical performance of 3D SnS_2_ QDs/rGO composite electrodes is further presented in Fig. 3g. The capacity of the pure SnS_2_ electrode drastically decreases to almost 16 mA/g after 200 cycles, while the 3D SnS_2_ QDs/rGO electrode can still maintain a value of 870 mAh/g after 200 cycles at a current density of 0.1 A/g. Moreover, in Fig. 3h, the test is performed to prove the better cycling performance of the composite at a scan rate of 0.5 A/g. After 200 charge/discharge cycles, a high reversible capacity of 622 mAh/g remained and the average Coulombic efficiency is as high as 99.44%.

To further understand the better cycle life of the 3D SnS_2_ QDs/rGO electrode, a TEM image is acquired to prove the distribution of the SnS_2_ QDs (in Additional file 1: Figure S3, by measuring 100 representative particles using the Nano-Measure software). The SnS_2_ QDs with ~ 6 nm are almost evenly anchored and limited within the rGO layers, indicating a strong adsorption between SnS_2_ QDs and the rGO layers. Overall, the results of both the electrochemical test and the particle distribution demonstrate that the introduction of rGO and the 3D honeycomb-like network offers abundant void spaces for volume expansion of SnS_2_ QDs. These structures act as channels for fast transportation of electron along all three directions and effectively restrain the aggregation. Thus, the rate performance and cycling stability of the composite are enhanced. Figure 3f shows a comparison of the electrochemical performance between the 3D SnS_2_ QDs/rGO composite (the current study) and the previously reported SnS_2_-based materials composite. It can be observed that the capacity of 3D SnS_2_ QDs/rGO in our study remains 862 mAh/g LIB at 0.1 A/g after 200 cycles, which is higher than the other rGO and SnS_2_-based material, such as graphene-SnS_2_ hybrids [39], acetylene black-SnS_2_ [40], SnS_2_@reduced graphene oxide [41], mesoporous carbon anchored with SnS_2_ nanosheets [42], graphene-SnS_2_ [43], SnS_2_ nanoparticle-loaded grapheme [44], SnS_2_@graphene [5], and Ultrathin SnS_2_ nanoparticles on graphene nanosheets [45].

**Fig. 4:**
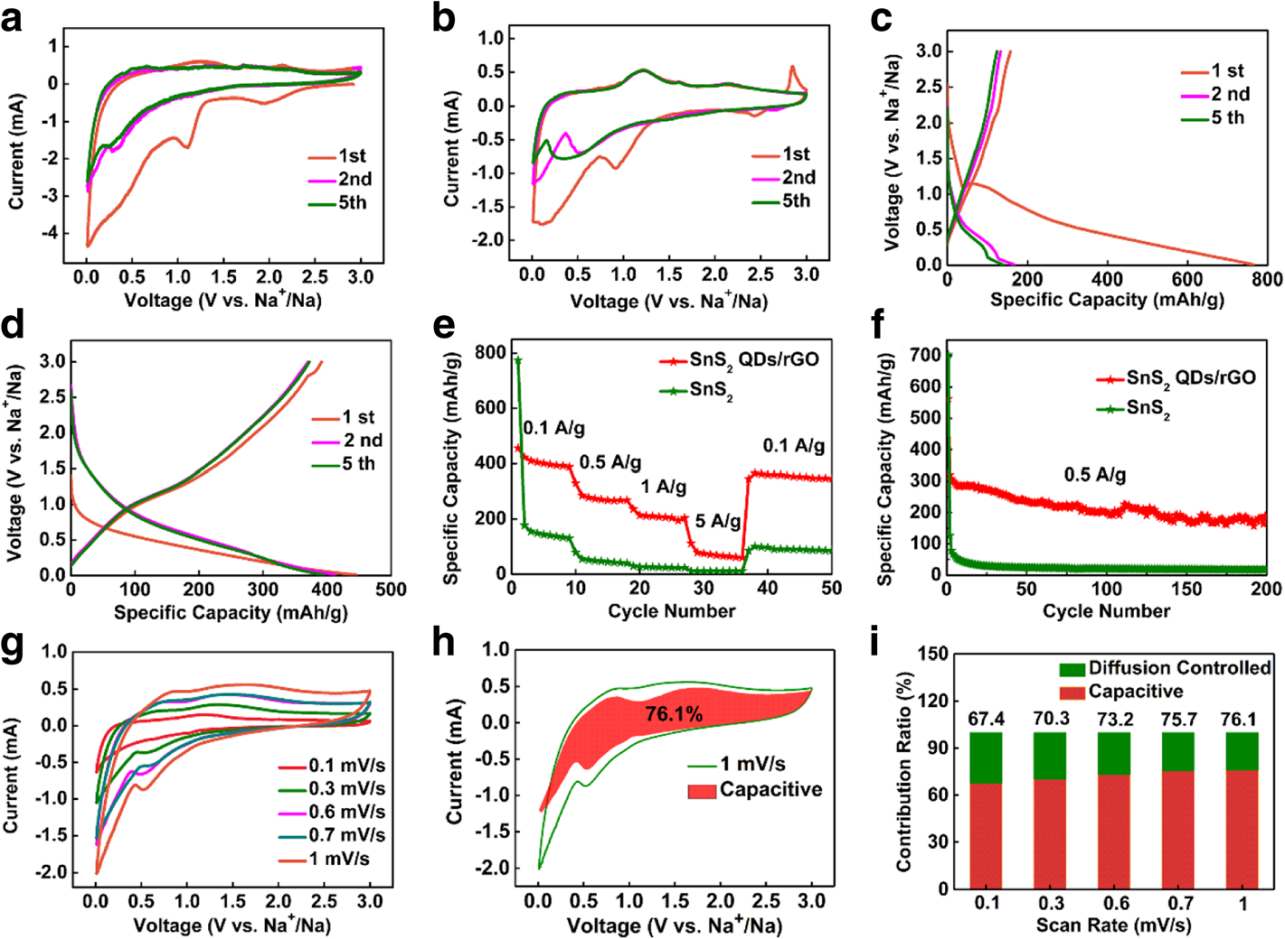
Electrochemical performance of the pure SnS_2_ and 3D SnS_2_ QDs/rGO composite electrodes for SIBs. **a**, **b** CV curves of the pure SnS_2_ electrode and 3D SnS_2_ QDs/rGO composite electrode at a scan rate of 0.1 mV/s for the first five cycles. **c** Charge/discharge curves of the pure SnS_2_ composite electrode and **d** the 3D SnS_2_ QDs/rGO composite electrode at a current density of 0.1 A/g in the voltage range of 0.01–3.0 V vs. Na^+^/Na. **e** Rate performance of 3D SnS_2_ QDs/rGO and the pure SnS_2_ composite electrode at rates ranging from 0.1 to 5 A/g. **f** Cycling performance of 3D SnS_2_ QDs/rGO composite electrode and the pure SnS_2_ electrode at a current density of 0.5 A/g. **g** CV curves of 3D SnS_2_ QDs/rGO composite electrode at different scan rates. **h** Capacitive contribution at the scan rate of 1 mV/s. **i** Contribution ratio of the capacitive and diffusion-controlled charge vs. different scan rates

To investigate the sodium storage processes of 3D SnS_2_ QDs/rGO and pure SnS_2_ as anode materials for SIBs, cyclic voltammetry is performed at a scan rate of 0.1 mV/s between 0.01 and 3.00 V vs Na^+^/Na, as shown in Fig. 4a, b. In Fig. 4a, the reduction peak at 0.3–1.0 V is corresponded to the conversion, alloying reactions (Eqs. (4) and (5)), and the formation of the SEI layer in the initial cycle. In Fig. 4b, the rather broad peak at ~ 1.0 V in the first reduction process is corresponded to the insertion of Na^+^ into the SnS_2_ layers (analogous to that of Li intercalation) according to Eqs. (3) [46, 47]:

3 $$ \mathrm{Sn}{\mathrm{S}}_2+\mathrm{x}\ {\mathrm{Na}}^{+}+{\mathrm{x}\mathrm{e}}^{-}\to {\mathrm{Na}}_{\mathrm{x}}\mathrm{Sn}{\mathrm{S}}_2 $$

4 $$ {\mathrm{Na}}_{\mathrm{x}}\mathrm{Sn}{\mathrm{S}}_2+\left(4-\mathrm{x}\right){\mathrm{Na}}^{+}+\left(4-\mathrm{x}\right){\mathrm{e}}^{-}\to \mathrm{Sn}+2{\mathrm{Na}}_2\mathrm{S} $$

5 $$ \mathrm{Sn}+\mathrm{x}{\mathrm{Na}}^{+}+\mathrm{x}{\mathrm{e}}^{-}\to {\mathrm{Na}}_{\mathrm{x}}\mathrm{Sn} $$

In reverse scanning, the unconspicuous oxidation peaks at 0.35, 1.2, and 2.25 V are ascribed to the desodiation of Na_x_Sn. The obvious oxidation peak at 1.2 V belongs to the resilience of the initial 3D SnS_2_ QDs/rGO electrode [25]. Note that the subsequent CV scans of 3D SnS_2_ QDs/rGO overlap well after the first cycle, indicating good reversibility of it for the sodiation and desodiation reactions.

The discharge-charge voltage profiles of the pure SnS_2_ and 3D SnS_2_ QDs/rGO electrodes are carried out between 0.01 and 3 V at a current density of 0.1 A/g. The corresponding charge/discharge profiles (1st, 2nd, and 5th cycles) are shown in Fig. 4c, d, respectively, which are in accord with the CV results. In Fig. 4c, a remarkable plateau appears at ~ 1.0 V in the discharge process, belonging to the formation of Na_x_SnS_2_. The plateau at 0.5–1.0 V is attributed to the conversion, while that below 0.5 V is assigned to the alloying reactions between Na^+^ and Sn. Then, the CV curve of the 3D SnS_2_ QDs/rGO electrode (Fig. 4d) indicates that the inconspicuous plateau voltage at ~ 1.0 V is assigned to the intercalation of Na^+^ into SnS_2_ layers during the first discharge process and this reaction is expressed as Eq. (3). The slope plateau at 0.3–1.0 V corresponds to the conversion reaction (Eq. (4)), the formation of the SEI layer by the irreversible insertion of Na^+^, and the decomposition of the electrolyte. The plateau below 0.3 V corresponds to the alloying reaction (Eq. (5)) [48–50]. The electrode shows a plateau at ~ 1.0 V and a slope plateau at ~ 1.6 V in the charge process, which are also in agreement with the CV results.

The rate capability of the pure SnS_2_ and 3D SnS_2_ QDs/rGO electrodes from 0.1 to 5 A/g in the SIB test are given in Fig. 4e. The 3D SnS_2_ QDs/rGO electrode is remarkably superior by comparison. It can be seen that the discharge capacities at the rate of 0.1, 0.5, 1, and 5 A/g are 397, 286, 213, and 95 mAh/g, respectively, and then easily return to 393 mAh/g at 0.1 A/g. But for the pure SnS_2_ electrode, the discharge capacity decays to 180, 59, 25, and 11 mAh/g with the discharge rate increased to 0.1, 0.5, 1, and 5 A/g, respectively. Then the discharge capacity only restores to 102 mAh/g when the discharge rate recovers to 0.1 A/g. The 3D SnS_2_ QDs/rGO electrode shows slight changes in discharge capacity after discharge at different current densities, which indicates better resilience of the nanostructure. Obviously, the unique 3D honeycomb-like structure allows Na^+^ transport at high current density without creating many irreversible changes of the electrode’s nanostructure, resulting in an excellent performance in SIBs. The discharge capacity of the pure SnS_2_ electrode retains only 6 mAh/g after 200 cycles at a scan rate of 0.5 A/g, which is significantly lower than that 233 mAh/g in the 3D SnS_2_ QDs/rGO electrode, as presented in Fig. 4f. A serious capacity decay of the pure SnS_2_ electrode can result from the low electronic conductivity of the unsupported SnS_2_ and the uncontrollable aggregations of Sn (or its discharge products) during the cycling. Thus, the outstanding electrochemical performance of the electrode corresponds to the 3D honeycomb-like structure. The existing porous in the structure can efficiently adjust the volume change in the process of alloying and de-alloying.

To better understand the charge storage process, the CV curves at various scan rates (0.1–1 mV/s) are performed to understand the electrochemical process (Fig. 4g). A peak shift appears with the scanning rate rising from 0.1 to 1 mV/s, indicating the polarization of the electrode. The capacity contribution from capacitive and diffusion-controlled charge can be quantified according to the relation [51] i (V) = k_1_v + k_2_v^1/2^, where k_1_v and k_2_v^1/2^ are the contributions from the capacitive and diffusion-controlled processes, respectively. From Fig. 4h, it can be observed that the capacitance-controlled capacity accounts for 76.1% of the total charge storage at a scan rate of 1 mV/s. With the scan rate increases in the order of 0.1, 0.3, 0.6, 0.7, and 1 mV/s, the proportion of the capacitance-controlled process increases from 67.4, 70.3, 73.2, 75.7, to 76.1%, respectively (Fig. 4i). The result indicates that the capacitive charge storage plays an important role in the total capacity of the electrode [52]. However, the relative rapid capacity decreases at higher scan rate is attributed to the diffusion-limited electrochemical energy conversion process [53].

**Fig. 5:**
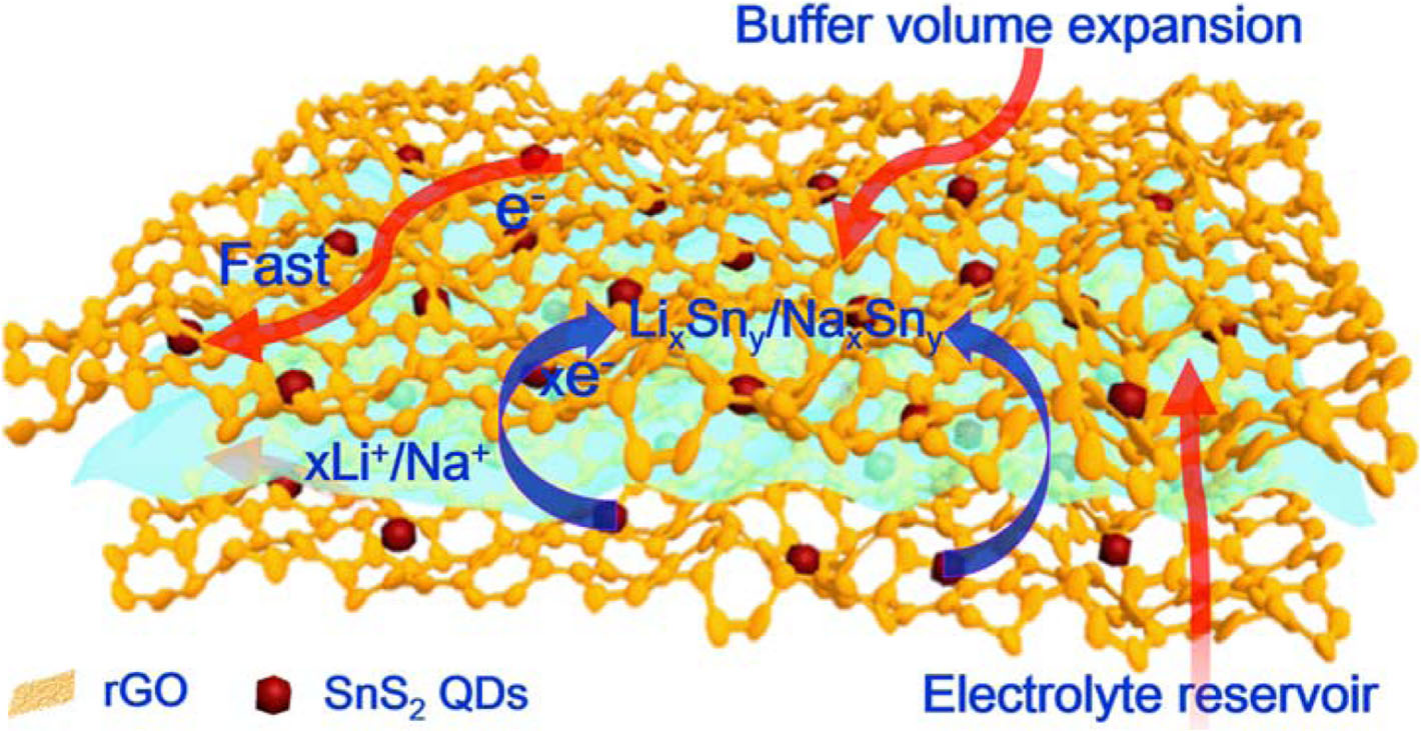
Schematic illustration highlights the structural benefits of the 3D SnS_2_ QDs/rGO composite during the charge/discharge process

The structural advantages of the 3D SnS_2_ QDs/rGO composite in LIBs and SIBs can be summarized as follows (Fig. 5): (i) the 3D structure can buffer the volume expansion and inhibit the agglomeration of SnS_2_ QDs during the charge/discharge process. (ii) The 3D honeycomb-like porous structure can provide sufficient space for electrolyte storage. (iii) The 3D interconnected network is beneficial for enhancing electron conductivity and allowing the electron to transfer quickly in the continuous paths. (iv) The SnS_2_ QDs with a particle size about ~ 6 nm can shorten the diffusion distance of Li^+^/Na^+^, resulting in good electrochemical performance.

## Conclusions

A novel 3D honeycomb-like SnS_2_ QDs/rGO composite was synthesized by one-pot spray drying and sulfidation. The SnS_2_ QDs (~ 6 nm) was uniformly distributed in the rGO layers. The thicknesses of the rGO sheets could be regulated by changing the concentration of GO in the spray solution. What is more, the size of the rGO nanovoids could be easily adjusted by using different size of the PS nanospheres. The 3D honeycomb-like rGO could not only buffer the volume expansion of the SnS_2_ QDs but also enhance their poor electrical conductivity. In addition, it can provide enough space for electrolyte reservoirs. As a result, the retention of the reversible capacity of the 3D SnS_2_ QDs/rGO electrode for LIB at 0.1 A/g was nearly 862 mAh/g and the capacity was as high as 622 mAh/g after 200 cycles at 0.5 A/g. Moreover, a capacity of 233 mAh/g could be delivered after 200 cycles at 0.5 A/g in the SIB test. The novel 3D honeycomb-like SnS_2_ QDs/rGO composite suggested a new strategy for preparing anode material in LIBs and SIBs. This advanced anode materials is predicable to have a significant influence on the energy storage field, and thus, provide fresh opportunities to enhance the electrochemical performance of Li^+^ and Na^+^ storage devices.

## Additional File

Additional file 1: **Figure S1.** a SEM image of the 3D SnO_2_ /rGO composite. b TEM image of the 3D SnO_2_ /rGO composite. c TEM image of the rGO backbone (after the removal of PS nanospheres). d TEM image of the rGO layer. **Figure S2.** a XRD pattern of the pure SnS_2_ composite. **Figure S3.** Particle size distribution of 3D SnS_2_ QDs/rGO after 200 charge/discharge cycles. **Figure S4a.** TGA curves of the 3D SnS_2_ QDs/rGO composite under air flow with a temperature ramp of 10 °C min^−1^ from 30 °C temperature to 800 °C. (DOCX 2376 kb)

## Abbreviations

3D SnS_2_ QDs/rGO: 3D Honeycomb-like SnS_2_ Quantum Dots/rGO
3D: Three-dimensional
BET: The Brunauer–Emmett–Teller
CV: Cyclic voltammetry
DEC: Diethyl carbonate
EC: Ethylene carbonate
GO: Graphene oxide
LIBs: Lithium-ion batteries
PS: Polystyrene
SEI: Solid electrolyte interface
SEM: Field emission scanning electron microscope
SIBs: Sodium-ion batteries
TEM: Transmission electron microscope
TGA: Thermogravimetric analyzer
XPS: X-ray photoelectron spectroscopy
XRD: X-ray diffraction
