# Supplementary material for: Rational Design of 3D Honeycomb-Like SnS2 Quantum Dots/rGO Composites as High-Performance Anode Materials for Lithium/Sodium-Ion Batteries
Source: Nanoscale Res Lett. 2018 Dec 3;13:389. doi: 10.1186/s11671-018-2805-x (PMC6277259; doi:10.1186/s11671-018-2805-x)
Supplement: Supplementary file 1 — Figure S1. a SEM image of the 3D SnO2 /rGO composite. b TEM image of the 3D SnO2 /rGO composite. c TEM image of the rGO backbone (after the removal of PS nanospheres). d TEM image of the rGO layer. Figure S2. a XRD pattern of the pure SnS2 composite. Figure S3. Particle size distribution of 3D SnS2 QDs/rGO after 200 charge/discharge cycles. Figure S4a. TGA curves of the 3D SnS2 QDs/rGO composite under air flow with a temperature ramp of 10 °C min−1 from 30 °C temperature to 800 °C. (DOCX 2376 kb) [file 11671_2018_2805_MOESM1_ESM.docx]

**Additional file 1**

**Rational Design of 3D Honeycomb-like SnS_2_ Quantum Dots/rGO Composites as High-performance Anode Materials for Lithium/Sodium ion Batteries**

Yingge Zhang,^a,b,^^[[1]](#footnote-1)^ Yan Guo,^a,b,1^Yange Wang,^a,b,1^ Tao Peng,^a,b^ Yang Lu,^a,b^ Rongjie Luo,^a,b^ Yangbo Wang,^a,b^ Xianming Liu,^*c^ Jang-Kyo Kim,^d^ and Yongsong Luo^*a,b^

*^a^School of Physics and Electronic Engineering, Xinyang Normal University, Xinyang 464000, P. R. China.*

*^^[[2]](#footnote-2)^b^Key Laboratory of Microelectronics and Energy of Henan Province, Xinyang Normal University, Xinyang 464000, P. R. China.*

*^c^College of Chemistry and Chemical Engineering, Luoyang Normal University, Luoyang 471022, P. R. China.*

*^d^Department of Mechanical and Aerospace Engineering, Hong Kong University of Science and Technology, Clear Water Bay, Kowloon, Hong Kong, P. R. China.*


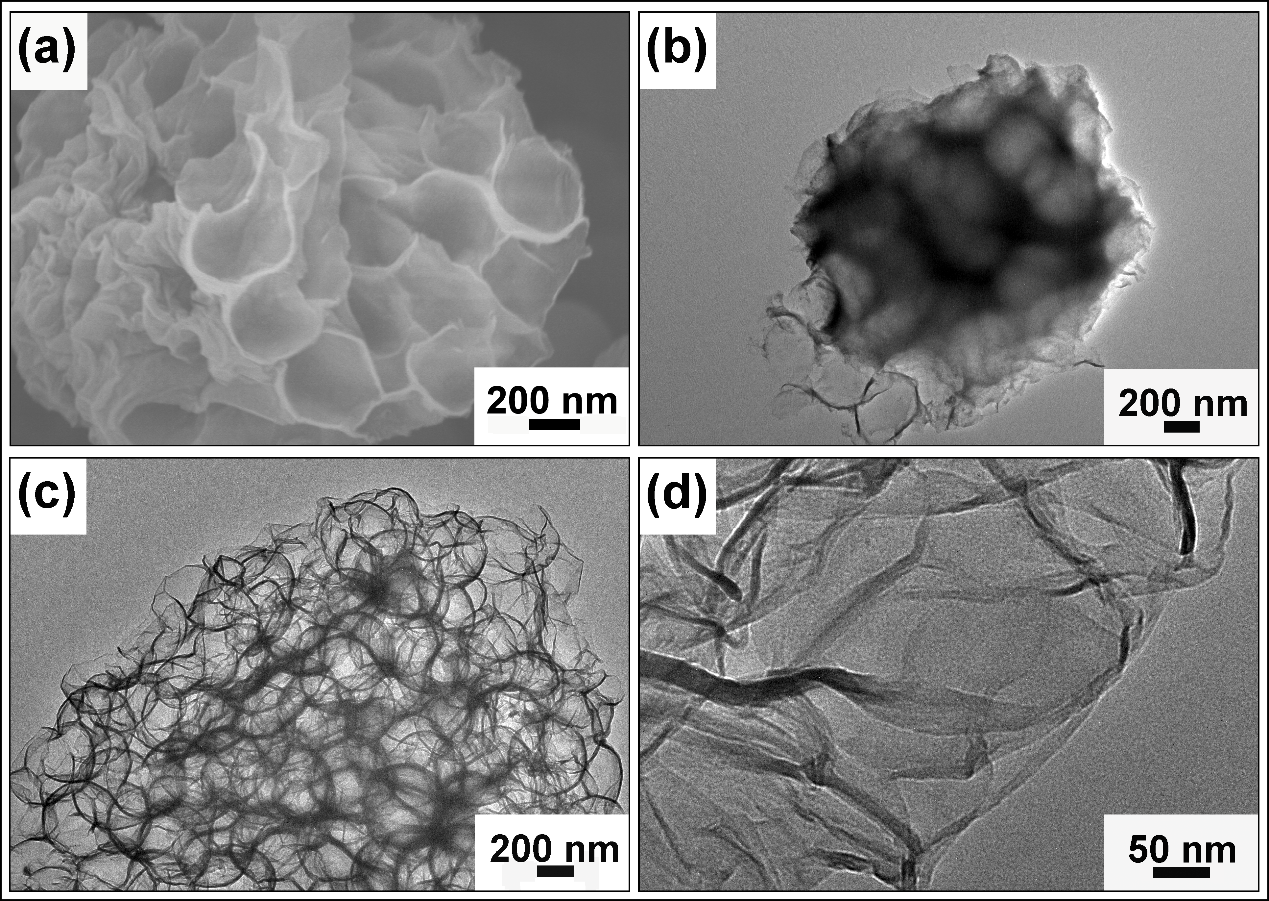


**Fig. S1**

**Fig. S1.** **a** SEM image of the 3D SnO_2_ /rGO composite. **b** TEM image of the 3D SnO_2_ /rGO composite. **c** TEM image of the rGO backbone (after the removal of PS nanospheres). **d** TEM image of the rGO layer.


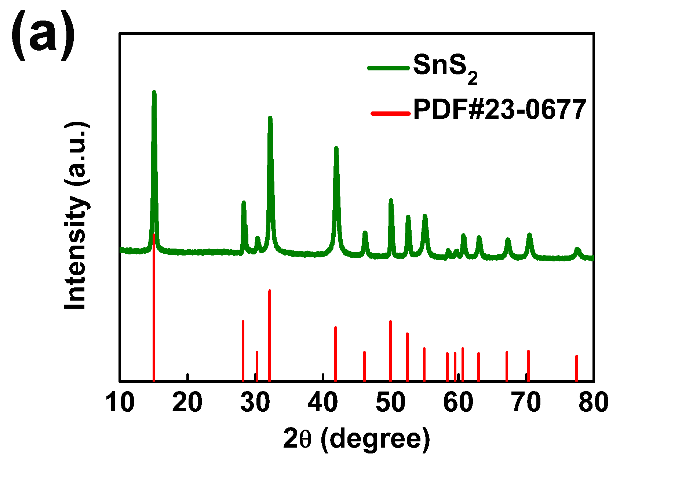


**Fig. S2**

**Fig. S2. a** XRD pattern of the pure SnS_2_ composite.

**
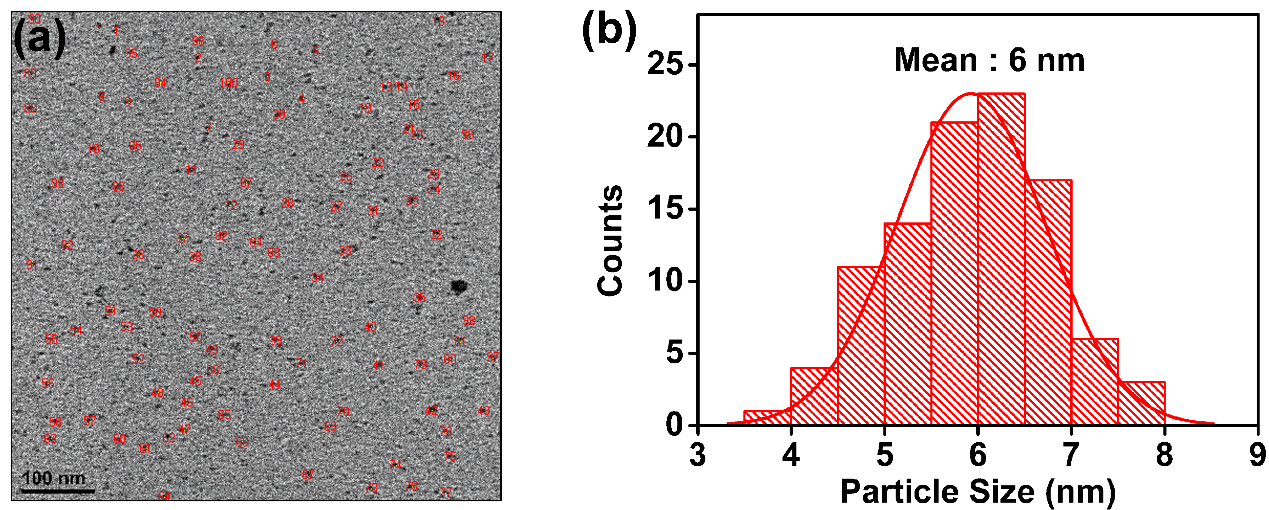
**

**Fig. S3**

**Fig. S3.** Particle size distribution of 3D SnS_2_ QDs/rGO after 200 charge/discharge cycles.





**Fig. S4a** TGA curves of the 3D SnS_2_ QDs/rGO composite under air flow with a temperature ramp of 10 ^o^C min^−1^ from 30 ^o^C temperature to 800 ^o^C.

1. These authors contributed equally to this work [↑](#footnote-ref-1)
2. * To whom correspondence should be addressed: Tel/fax: +86 376 6390801, E-mail: [ysluo@xynu.edu.cn](mailto:ysluo@xynu.edu.cn) (Y. S. Luo). [myclxm@163.com (X](mailto:myclxm@163.com (X). M. Liu). [↑](#footnote-ref-2)
